# Long-Term MALT1 Inhibition in Adult Mice Without Severe Systemic Autoimmunity

**DOI:** 10.1016/j.isci.2020.101557

**Published:** 2020-09-12

**Authors:** Annelies Demeyer, Yasmine Driege, Ioannis Skordos, Julie Coudenys, Kelly Lemeire, Dirk Elewaut, Jens Staal, Rudi Beyaert

**Affiliations:** 1 Center for Inflammation Research, VIB, Technologiepark-Zwijnaarde 71, 9052 Ghent, Belgium; 2 Department of Biomedical Molecular Biology, Ghent University, Technologiepark-Zwijnaarde 71, 9052 Ghent, Belgium; 3 Department of Internal Medicine and Pediatrics, Ghent University, Technologiepark-Zwijnaarde 71, 9052 Ghent, Belgium

**Keywords:** Immunology, Cell Biology

## Abstract

The protease MALT1 is a key regulator of NF-κB signaling and a novel therapeutic target in autoimmunity and cancer. Initial enthusiasm supported by preclinical results with MALT1 inhibitors was tempered by studies showing that germline MALT1 protease inactivation in mice results in reduced regulatory T cells and lethal multi-organ inflammation due to expansion of IFN-γ-producing T cells. However, we show that long-term MALT1 inactivation, starting in adulthood, is not associated with severe systemic inflammation, despite reduced regulatory T cells. In contrast, IL-2-, TNF-, and IFN-γ-producing CD4^+^ T cells were strongly reduced. Limited formation of tertiary lymphoid structures was detectable in lungs and stomach, which did not affect overall health. Our data illustrate that MALT1 inhibition in prenatal or adult life has a different outcome and that long-term MALT1 inhibition in adulthood is not associated with severe side effects.

## Highlights

• Inducible MALT1 inactivation for up to 6 months in the absence of severe toxicity
• MALT1 inactivation in adult mice decreases Tregs without effector T cell activation
• Long-term MALT1 inactivation results in tertiary lymphoid structure formation
• MALT1 inhibition in prenatal or adult life has a different outcome

## Graphical Abstract

**Figure:**
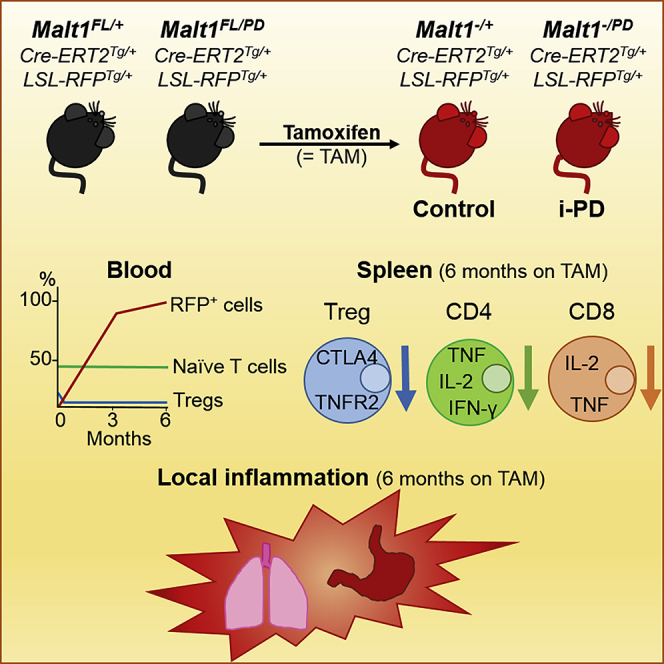

## Introduction

The paracaspase MALT1 is an important player in innate and adaptive immune signaling (Ruland and Hartjes, 2019). MALT1 is best known for its role in T cell receptor (TCR) signaling leading to nuclear factor (NF)-κB-dependent gene expression, which mediates the activation and proliferation of conventional T cells as well as the development of regulatory T cells (Tregs). Upon TCR triggering, MALT1 is activated as part of the so-called CARD11/BCL10/MALT1 (CBM) complex, where MALT1 functions as a scaffolding protein to recruit the E3 ubiquitin ligase TRAF6 and activate the IκB kinase complex, leading to NF-κB activation (Sun et al., 2004; Oeckinghaus et al., 2007; Lork et al., 2019). TCR engagement also activates MALT1 proteolytic activity, leading to the cleavage of specific substrates (Ruland and Hartjes, 2019). Some of these MALT1 substrates, such as the deubiquitinases A20 and CYLD (Staal et al., 2011; Coornaert et al., 2008), and the mRNA-destabilizing proteins Regnase-1, Roquin-1, and Roquin-2 (Jeltsch et al., 2014; Uehata et al., 2013) are well-known critical negative regulators of pro-inflammatory gene expression whose cleavage is believed to further fine-tune TCR-induced gene expression. Importantly, inhibition of MALT1 enzymatic activity does not influence its scaffolding function, and downstream IκB kinase activation is therefore not affected (Bardet et al., 2018). In fact, specific inhibition of MALT1 enzymatic activity revealed a unique transcriptional fingerprint of MALT1 protease activity, indicating that the proteolytic activity of MALT1 can regulate signaling pathways well beyond NF-κB (Bardet et al., 2018).

MALT1 proteolytic activity is essential to drive T cell survival and expansion (Bardet et al., 2018). Similarly, MALT1 proteolytic activity is essential for the survival and proliferation of certain cancer cells, including ABC-type diffuse large B cell lymphoma and mantle cell lymphoma (Juilland and Thome, 2016). Moreover, MALT1 inhibition may also indirectly decrease tumor growth by interfering with the immune-suppressive function of Tregs (Di Pilato et al., 2019; Rosenbaum et al., 2019). Therefore, the therapeutic potential of MALT1 inhibition in autoimmune disease and cancer has raised a lot of interest and led to the development of potent small compound MALT1 inhibitors (Bardet et al., 2018). Several preclinical studies already showed a protective effect of pharmacological MALT1 inhibition in murine models of multiple sclerosis (Mc Guire et al., 2014), arthritis (Martin et al., 2020b), lymphoma (Fontan et al., 2012; Nagel et al., 2012; Dai et al., 2017; Jacobs et al., 2020), and glioblastoma (Jacobs et al., 2020). Noteworthy, a first-in-human phase I clinical study in participants with relapsed/refractory B-cell non-Hodgkin lymphoma and chronic lymphocytic leukemia has recently been initiated (https://clinicaltrials.gov/ct2/show/NCT03900598). A major concern was, however, raised by several independent studies showing that knock-in mice constitutively expressing a catalytically inactive MALT1 “protease-dead” mutant (*Malt1*-PD mice) rapidly develop lethal autoimmune inflammation in multiple organs (Gewies et al., 2014; Jaworski et al., 2014; Yu et al., 2015; Bornancin et al., 2015; Demeyer et al., 2019; Martin et al., 2019). This was somewhat surprising because a previous study had shown that pharmacological inhibition of MALT1 in mice for up to 3 weeks does not cause any side effects (Mc Guire et al., 2014). One major difference, however, between the genetic models and inhibitor treatment is that germline *Malt1*-PD mice lack protease activity at conception or at a very young age, whereas MALT1 inhibitor treatment is done in adult life. In this context, the severe reduction in thymic Tregs in *Malt1*-PD mice, which are mostly formed at young age (Fontenot et al., 2005), has been suggested to be at the origin of the severe autoimmune phenotype of these mice (Jaworski et al., 2014; Gewies et al., 2014; Demeyer et al., 2019; Baens et al., 2018). However, recent studies showed that MALT1 protease activity is also critical for maintaining Treg function (Cheng et al., 2019; Rosenbaum et al., 2019), implicating a risk for autoimmunity when MALT1 protease activity is only lost in adulthood. To have a clear view on the safety of long-term inhibition of MALT1 in adults, we have therefore generated a full-body tamoxifen-inducible *Malt1*-i-PD mouse model, which allowed us to monitor the effect of MALT1 inactivation in adult mice for up to 6 months.

## Results and Discussion

### Long-Term Inducible MALT1 Inactivation in Adult Mice Decreases Tregs Without Increasing Effector T Cell Activation

**Figure 1:**
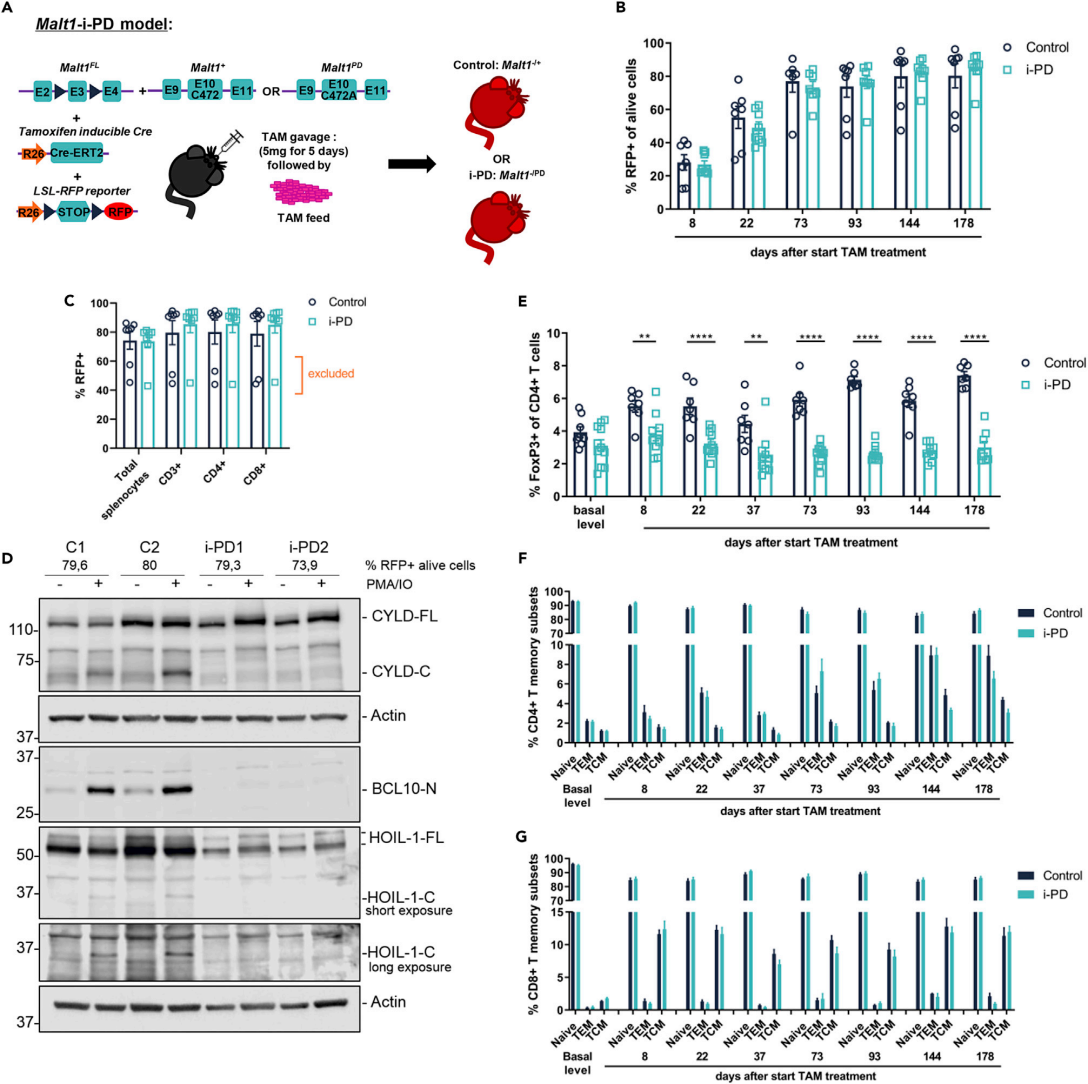
Long-Term Inducible MALT1 Protease Inactivation Reduces Circulating Treg Levels without Changing the Activation Status of CD4^+^ and CD8^+^ T Cells (A) Strategy to generate *Malt1*-i-PD mice. (B) Frequency of RFP^+^ alive cells in blood of *Malt1*-i-PD and control mice at several time points after starting tamoxifen treatment. (C) Frequency of living RFP^+^ splenocytes, CD3^+^, CD4^+^, and CD8^+^ splenic T cells in *Malt1*-i-PD and control mice after 6 months tamoxifen treatment. Excluded = 3 mice with less than 70% RFP^+^ alive splenocytes that were excluded for subsequent analysis. (D) Immunoblot for CYLD, HOIL-1, and cleaved BCL10 on splenocyte extracts of control (C1 and C2) and two different *Malt1*-i-PD mice (i-PD1 and i-PD2) stimulated *in vitro* with PMA and ionomycin (PMA/IO) for 1.5 h to activate MALT1. Frequency of RFP^+^ splenocytes of these mice is indicated. (E–G) (E) Frequency of Tregs; (F) naive, effector memory (TEM) and central memory (TCM) CD4^+^ T cells; and (G) CD8^+^ T cells in blood before and at several time points after starting tamoxifen treatment. (B, C, and E–G) Data (control: n = 7 and *Malt1*-i-PD: n = 8) obtained by flow cytometry, presented as mean ± SEM and statistical significance: unpaired two-tailed Student's t test (∗∗p < 0.01; ∗∗∗∗p < 0.0001). All data are representative for two independent experiments.

To obtain a full-body tamoxifen-inducible *Malt1*-i-PD mouse model, we first generated *Malt1*^*PD/FL*^ or *Malt1*^*+/FL*^ mice with a tamoxifen-inducible Cre-ERT2 in one *Rosa26* allele (Hameyer et al., 2007) and a LoxP-stop-LoxP (LSL)-RFP reporter for Cre activity in the other *Rosa26* allele (Luche et al., 2007) (Figure 1A). Upon tamoxifen administration, the floxed third exon of the *Malt1*^*FL*^ allele and the floxed stop cassette of the RFP reporter are removed by Cre-mediated recombination. In this way we obtained *Malt1*^*+/−*^ (= control) and *Malt1*^*PD/-*^ (= *Malt1*-i-PD) mice with RFP^+^ cells representing cells in which Cre-ERT2 was active. Mice received tamoxifen for 5 days via oral gavage and were then kept on tamoxifen containing feed to prevent repopulation by *Malt1*^*+/FL*^ or *Malt1*^*PD/FL*^ bone marrow-derived cells. To monitor the efficiency of Cre recombination, RFP expression was assessed in blood cells at several time points following the start of tamoxifen treatment. After 2–3 months the majority of mice had 70% to 85% RFP^+^ blood cells (Figure 1B). *Malt1*-i-PD mice were sacrificed after 6 months tamoxifen treatment, and most mice had up to 90% or more CD3^+^ RFP^+^ cells in their spleen at this endpoint (Figure 1C). Mice showing less than 70% RFP^+^ cells were excluded from the analysis in subsequent experiments. MALT1 protease deficiency in *Malt1*-i-PD mice could be confirmed by the lack of CYLD, BCL10, and HOIL-1 cleavage in phorbol 12-myristate 13-acetate (PMA)/ionomycin-stimulated splenocytes (Figure 1D).

**Figure 2:**
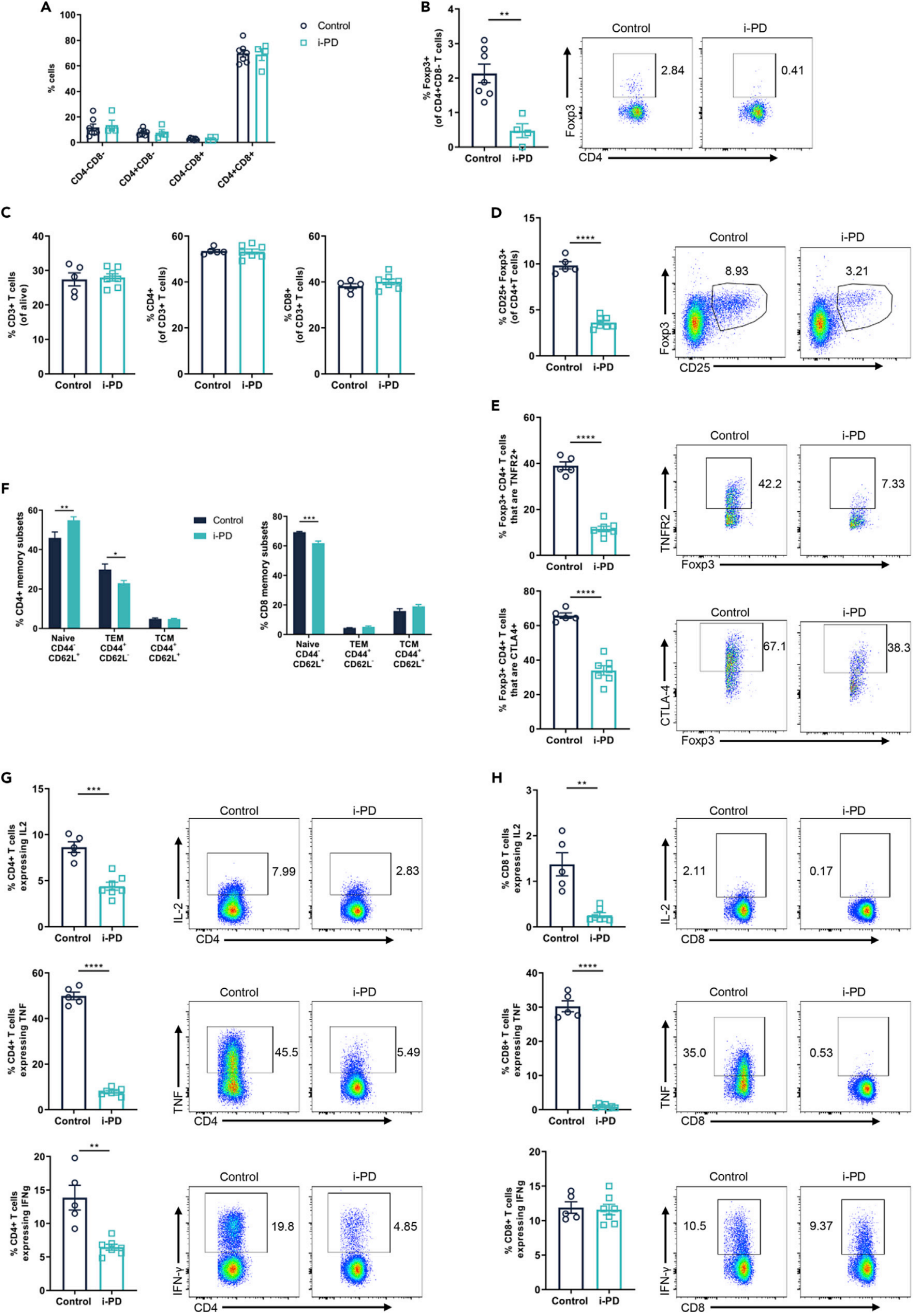
Long-Term Inducible MALT1 Protease Inactivation Reduces Thymic and Splenic Treg Levels Without Changing the Activation Status of Splenic CD4^+^ and CD8^+^ T Cells *Malt1*-i-PD (n = 4) and control (n = 7) mice were treated with tamoxifen for 6 months, and thymocytes were analyzed at endpoint by flow cytometry. (A and B) Frequency of (A) CD4^-^CD8^-^, CD4^+^CD8^-^, CD4^-^CD8^+^, and CD4^+^CD8^+^ cells and (B) Tregs (Foxp3^+^CD4^+^CD8^-^ T cells). *Malt1*-i-PD (n = 6) and control (n = 5) mice were treated with tamoxifen for 6 months, and splenocytes were analyzed at endpoint by flow cytometry. (C–H) (C) Frequency of CD3^+^, CD4^+^, and CD8^+^ T cells; (D) Tregs (CD25^+^Foxp3^+^CD4^+^ T cells); (E) Tregs expressing TNFR2 or CTLA4; (F) naive, TEM, TCM CD4^+^, and CD8^+^ T cells; (G) CD4^+^ T cells expressing IL-2, TNF, or IFN-γ; and (H) CD8^+^ T cells expressing IL-2, TNF, or IFN-γ. Data presented as mean ± SEM and statistical significance: unpaired two-tailed Student's t test (∗p < 0.05; ∗∗p < 0.01; ∗∗∗p < 0.001; ∗∗∗∗p < 0.0001). Representative flow cytometry plots showing gating strategy and individual cell frequencies are included. All data are representative for two independent experiments.

**Figure 3:**
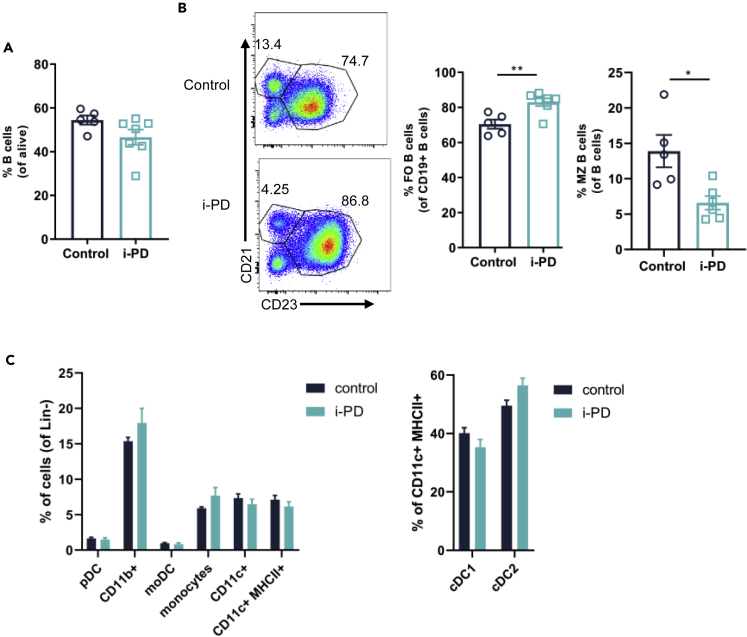
Long-Term Inducible MALT1 Protease Inactivation Reduces MZ B Cell Levels and has No Effect on Monocytes and Different DC Populations *Malt1*-i-PD (n = 7) and control (n = 5) mice were treated with tamoxifen for 6 months, and splenocytes were analyzed at endpoint by flow cytometry. (A–C) Frequency of (A) total B cells, (B) FO B cells and MZ B cells, and (C) monocytes and different DC populations. Data obtained by flow cytometry, presented as mean ± SEM and statistical significance: unpaired two-tailed Student's t test (∗p < 0.05; ∗∗p < 0.01). Representative flow cytometry plots showing gating strategy and individual B cell frequencies are included in (B). All data are representative for two independent experiments.

Germline inactivation of MALT1 protease activity in *Malt1*-PD mice is associated with a severe reduction of Tregs and activation of CD4^+^ and CD8^+^ T cells, leading to multi-organ inflammation (Jaworski et al., 2014; Gewies et al., 2014; Bornancin et al., 2015; Demeyer et al., 2019). In our study, *Malt1*-i-PD circulating Treg levels were already decreased upon 1 week tamoxifen treatment and reached about 30% of the levels present in control mice after 6 months (Figure 1E). However, despite this significant drop in Tregs, blood CD4^+^ and CD8^+^ T cell activation remained unaltered between control and *Malt1*-i-PD mice (Figures 1F and 1G). At endpoint, *Malt1*-i-PD and control mice had similar amounts of thymic and splenic CD4^+^ and CD8^+^ T cells (Figures 2A and 2C). However, the amount of thymic Tregs and splenic Tregs had dropped to 25% and 30%, respectively, compared with control mice (Figures 2B and 2D). Also, Treg expression of the functionality markers CTLA4 and TNFR2 was severely reduced upon MALT1 inactivation (Figure 2E), which is in agreement with the described role of MALT1 protease activity in TCR-induced CTLA4 expression in Tregs (Rosenbaum et al., 2019). Importantly, the significant drop in CTLA4- and TNFR2-expressing splenic Tregs in *Malt1*-i-PD mice did not result in overt splenic CD4^+^ and CD8^+^ T cell activation (Figure 2F). Moreover, the number of TNF-, IL-2-, and IFN-γ-producing CD4^+^ T cells was strongly reduced upon long-term MALT1 inactivation (Figure 2G). Similar results were obtained for CD8^+^ T cells, with the surprising exception for IFN-γ-producing CD8^+^ T cells, which were not altered (Figure 2H). Importantly, the reduction in IFN-γ-producing CD4^+^ T cells upon long-term inducible MALT1 inactivation is in strong contrast to the increase in IFN-γ-producing CD4^+^ T cells described in constitutive germline *Malt1-*PD mice (Jaworski et al., 2014; Demeyer et al., 2019; Martin et al., 2019). Total B cell levels were similar at endpoint (Figure 3A), but the frequency of MZ B cells was reduced in *Malt1*-i-PD mice (Figure 3B), which is similar to the previously described drop in *Malt1*-PD mice (Demeyer et al., 2019; Gewies et al., 2014; Bornancin et al., 2015; Yu et al., 2015; Jaworski et al., 2014; Baens et al., 2018). There were no significant differences in the amount of monocytes and different DC populations between control and *Malt1*-i-PD mice (Figure 3C). Together, these results clearly show that long-term inducible inactivation of MALT1 protease activity in adult mice mainly affects the lymphocyte compartment of the immune system. Most importantly and in contrast to constitutive MALT1 protease inactivation by germline engineering, long-term MALT1 protease inactivation in adult mice does not lead to the activation of CD4^+^ and CD8^+^ T cells in blood or spleen, despite significantly reduced Treg levels.

### Long-Term Inducible MALT1 Inactivation in Adult Mice Does Not Cause Systemic Inflammation

**Figure 4:**
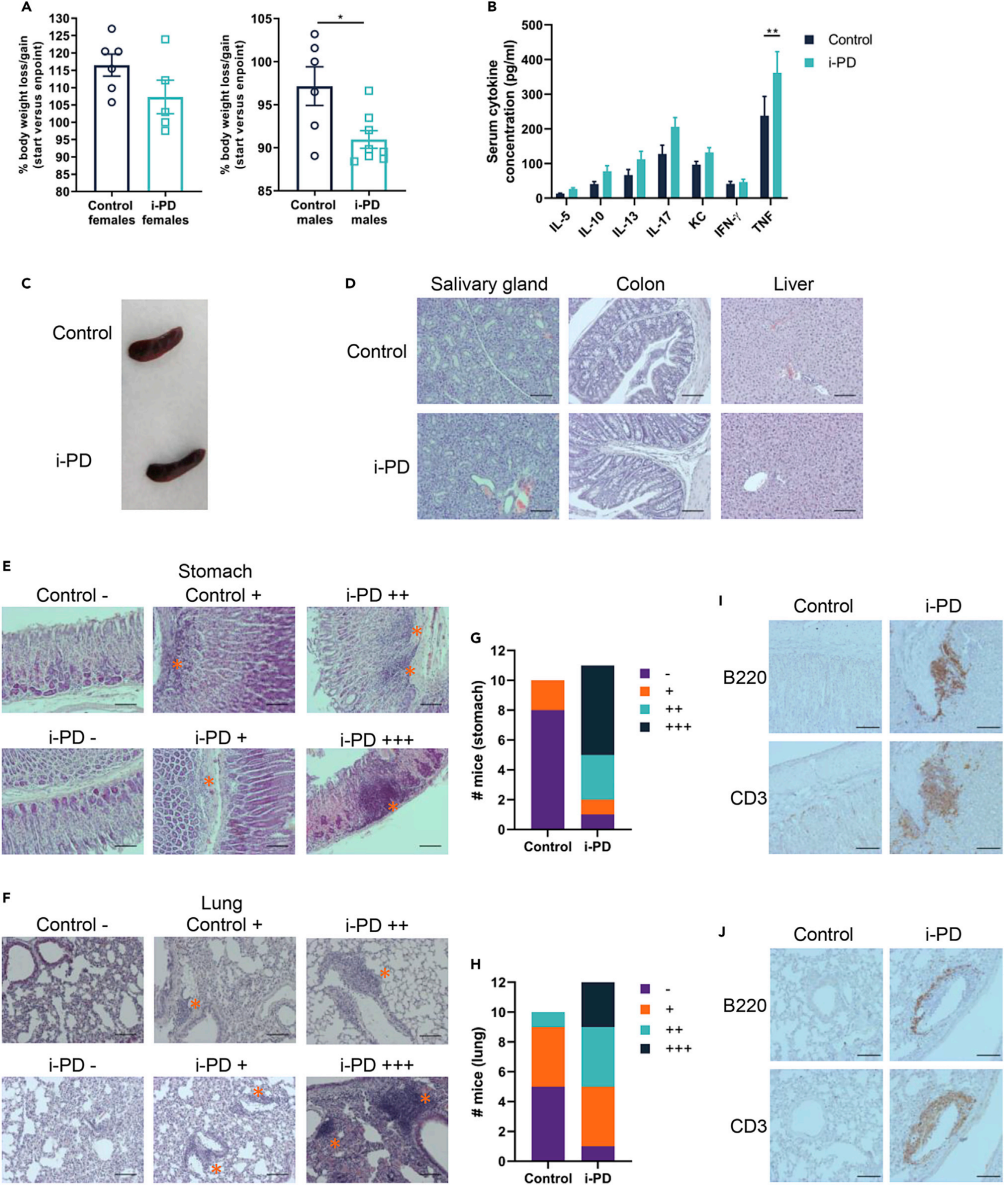
Long-Term Inducible MALT1 Protease Inactivation is Associated with Local Immune Cell Infiltration in Stomach and Lungs *Malt1*-i-PD and control mice were treated with tamoxifen for 6 months, and analyzed at endpoint. (A) Change in body weight of female (control: n = 6 and *Malt1*-i-PD: n = 5) and male (control: n = 6 and *Malt1*-i-PD: n = 8) mice at endpoint compared with their initial weight. (B) Serum cytokine concentration of *Malt1*-i-PD (n = 12) and control (n = 11) mice. (C) Representative picture of the spleen of control and *Malt1*-i-PD mice. (D) Representative picture of H&E staining of salivary gland (control: n = 10 and *Malt1*-i-PD: n = 11), colon (control: n = 6 and *Malt1*-i-PD: n = 6), and liver (control: n = 10 and *Malt1*-i-PD: n = 12). (E and F) (E) Representative picture of different degrees of immune cell infiltration based on H&E staining of stomach and (F) lung from different control (n = 10) and *Malt1*-i-PD (n = 11) mice. Different degrees of immune cell infiltration (indicated with an asterisk): none (−), minor dispersed infiltration (+), multiple large infiltrations (++), and infiltrations resembling tertiary lymphoid organs (+++). (G and H) (G) Number of *Malt1*-i-PD and control mice showing the indicated degrees of stomach and (H) lung immune cell infiltration. (I and J) (I) Representative picture of anti-B220 and anti-CD3 staining of stomach (n = 3) and (J) lung (n = 3) tissue sections from control and *Malt1-*i-PD mice with infiltrations resembling tertiary lymphoid organs (+++). Scale bars, 100 μm in (D–F) and (I–J); (A and B) mean ± SEM and statistical significance: unpaired 2-tailed Student's t test (∗p < 0.05; ∗∗p < 0.01). All data are representative for two independent experiments. See also Figure S1.

In contrast to the severe and rapid body weight loss that was previously observed in constitutive *Malt1*-PD mice (Demeyer et al., 2019; Yu et al., 2015; Jaworski et al., 2014; Gewies et al., 2014; Bornancin et al., 2015), only a mild reduction in body weight was observed in male, but not female, *Malt1*-i-PD mice at endpoint (Figure 4A). Also, *Malt1*-i-PD mice did not develop severe ataxia that was previously observed in constitutive *Malt1*-PD mice. In addition, whereas constitutive *Malt1*-PD mice were reported to have high serum IFN-γ and TNF levels (Gewies et al., 2014; Demeyer et al., 2019), *Malt1*-i-PD mice only showed a very mild increase in TNF serum levels and no significant difference for IFN-γ, IL-5, IL-10, IL-13, IL-17, and KC (Figure 4B). Jaworski et al. reported IgE and IgG1 autoantibodies reactive against stomach antigens in constitutive *Malt1*-PD mice (Jaworski et al., 2014), whereas Martin et al. reported elevated IgE and IgG1 antibodies reactive against bacterial and food antigens (Martin et al., 2019), although these were not essential for the development of autoimmune inflammation in *Malt1*-PD mice. In our study, no autoantibodies against specific nuclear and cytoplasmic antigens could be detected in serum from *Malt1*-i-PD mice (Figure S1). Together, these results clearly show that, in contrast to constitutive MALT1 protease inactivation by germline engineering, long-term inducible inactivation of MALT1 protease activity in adult mice does not cause systemic inflammation, which is consistent with the absence of effector T cell activation.

### Long-Term Inducible MALT1 Inactivation in Adult Mice Induces Tertiary Lymphoid Structures in Specific Tissues

Constitutive *Malt1*-PD mice rapidly develop lethal multi-organ inflammation (Jaworski et al., 2014; Gewies et al., 2014; Bornancin et al., 2015; Demeyer et al., 2019; Martin et al., 2019). In contrast, *Malt1*-i-PD mice looked healthy after 6 months MALT1 inactivation. Spleen size was similar in both *Malt*1-i-PD and control mice (Figure 4C), and macroscopic examination of several other organs also did not show any clinical signs of disease. H&E-stained tissue sections did not reveal inflammation in salivary gland, colon, and liver (Figure 4D). However, almost all *Malt1*-i-PD mice suffered from increased immune cell infiltration in the stomach (Figures 4E and 4G) and lungs (Figures 4F and 4H). It should be mentioned that some control mice also displayed minor immune cell infiltration in stomach and even more in lungs, which may reflect their relatively advanced age. Some of the immune cell infiltrates in *Malt1*-i-PD mice resembled tertiary lymphoid structures (Pipi et al., 2018), which was further supported upon staining for B (B220) and T cells (CD3) (Figures 4I and 4J). Especially in lungs, a separate B cell zone, which is characteristic for inducible bronchus-associated lymphoid tissue formation (Woodland and Randall, 2004), could be observed (Figure 4J). It should be mentioned that stomach and lung inflammation has been described in the case of constitutive *Malt1*-PD mice (Jaworski et al., 2014; Gewies et al., 2014; Bornancin et al., 2015; Yu et al., 2015; Demeyer et al., 2019). Moreover, mice with a Treg-specific loss of MALT1 protease function also suffer from lung inflammation (Cheng et al., 2019). Together, these data demonstrate that, in contrast to the lethal multi-organ inflammation observed upon germline MALT1 protease inactivation, long-term inducible inhibition of MALT1 protease activity in adult mice is only associated with local immune cell infiltration in stomach and lungs.

In conclusion, our data indicate that long-term inhibition of MALT1 proteolytic activity in adult life is relatively safe and does not lead to destructive autoimmune inflammation as previously described upon germline inactivation of MALT1. These discrepancies most likely reflect the known key role of MALT1 proteolytic activity in thymic Treg development early during life, whereas peripheral Treg generation has been shown to be less MALT1 dependent (Brustle et al., 2015). In addition, MALT1 protease inhibition results in reduced cytokine production by T cells in the periphery, which might lead to a new equilibrium between residual Tregs on one side and lower cytokine-producing T cells on the other side. There is still a caveat of potential local autoimmunity in lungs and stomach upon MALT1 inhibition, which may reflect a specific role for MALT1 in immune tolerance toward locally displayed antigens. Interestingly, a recent study in rat and dog (Martin et al., 2020b) reported decreased Treg numbers and immune infiltration in specific organs, but no ataxia and lethal autoimmunity upon prolonged treatment with a novel MALT1 inhibitor (MLT-943), which is in line with our data. Nevertheless, despite an excellent selectivity profile of the used MALT1 inhibitor, it cannot be fully excluded that off-target effects of the inhibitor contribute to the observed side effects in the study of Martin et al. Our data with *Malt1*-i-PD mice, which do not suffer from off-target effects and show that prolonged and specific MALT1 targeting is only associated with mild immune cell infiltration in certain organs (stomach and lungs), therefore indicate that studies using different classes of compounds with improved specificity deserve further evaluation. Going forward, the evaluation of side effects of different classes of small compound inhibitors of MALT1 for the treatment of autoimmune inflammation or cancer should therefore especially focus on these tissues that show inflammation in our mouse model and in the models used by Martin et al. Similar immune-related adverse effects have been described for immune checkpoint inhibitors targeting CTLA4 (Friedman et al., 2016; Johncilla et al., 2019), which could be related to the observed decrease in CTLA4-expressing Tregs upon inducible MALT1 inhibition. Management strategies developed for those side effects of immune checkpoint inhibition could potentially also be useful to mitigate the risks of MALT1 protease inhibition (Brahmer et al., 2018; Friedman et al., 2016). The here-described inducible mouse model for MALT1 targeting is unique, in the sense that it allows to follow-up mice for a much longer time than possible with small compound MALT1 inhibitors that need daily treatment. Such prolonged studies are essential to increase the successful translation of preclinical mouse studies to patients. While the original enthusiasm of big pharmaceutical companies in therapeutic targeting of MALT1 was strongly tempered by multiple studies reporting a severe autoimmune phenotype of constitutive *Malt1*-PD mice, the results described here can be expected to stir a renewed interest in the development of small compound MALT1 inhibitors for several autoimmune diseases and cancers.

### Limitations of the Study

Lack of lethal autoimmunity in our *Malt1*-i-PD mice could still be due to the incomplete inactivation of MALT1. However, partial MALT1 inactivation in our mice mimics pharmacological targeting (Martin et al., 2020a). Conceptually our model is therefore very relevant in the context of therapeutic targeting of MALT1 with small compound inhibitors. Importantly, partial inactivation is what one also observes with most small compounds *in vivo*, exposure of which does not result in a complete and continuous inhibition due to the pharmacokinetic profile of the compound. In addition, there are several partial allosteric inhibitors that only show partial inhibition, known as maximal efficacy. This is very well known in the case of GPCRs (Verespy et al., 2016), but also exists for other enzymes such as proteases (Conn et al., 2009). It should be stressed that 2 to 3 months after the start of tamoxifen treatment, the number of RFP^+^ cells in the blood is for most mice between 70% and 85% (Figure 1B). Moreover, several mice included for endpoint analysis after 6 months had up to 90% RFP^+^ T cells (Figure 1C). (The level of inhibition in T cells is the most important because disease development in *Malt1*-PD mice was previously shown to be driven by defective MALT1 activity in T cells, Demeyer et al., 2019). Moreover, the above level of MALT1 inactivation clearly reduced the number of IL-2-, TNF-, and IFN-γ-producing CD4^+^ T cells (Figure 2G), which is known to be dependent on MALT1 proteolytic activity (Bardet et al., 2018). Last but not least, MALT1-dependent CYLD, HOIL-1, and BCL10 cleavage was completely abolished in splenocytes isolated from *Malt1*-i-PD mice with more than 70% RFP^+^ cells (Figure 1D). Together, these data illustrate that the obtained level of MALT1 inactivation is sufficient to prevent a MALT1-dependent biological response. In future studies, it will be of interest to determine the *in vivo* impact of this specific inhibition of MATL1 protease activity in Malt1-i-PD mice in an autoimmune disease model.

For our experiments we used specific pathogen-free (SPF) mice that might have a different immune status compared with mice housed in a conventional environment. However, a conventional mouse facility suffers from several unknown environmental factors that differ from laboratory to laboratory, which can seriously affect results and hamper reproducibility. SPF conditions have therefore been the standard in most mouse facilities, including ours, for many years. Moreover, we wanted to compare the results of *Malt1*-i-PD mice with previously reported data with constitutive *Malt1*-PD mice that were kept in an SPF facility. Importantly, although both mouse lines were kept in SPF conditions, we could show that *Malt1*-i-PD mice do not phenocopy the severe lethal autoimmunity in *Malt1*-PD mice.

### Resource Availability

#### Lead Contact

Further information and requests for resources and reagents should be directed to and will be fulfilled by the Lead Contact, Rudi Beyaert (Rudi.Beyaert@irc.vib-ugent.be).

#### Materials Availability

All mouse lines and reagents generated in this study are available from the Lead Contact with a completed Materials Transfer Agreement.

#### Data and Code Availability

This study did not generate large-scale datasets. Raw data of this article are available from the lead contact upon request.

## Methods

All methods can be found in the accompanying Transparent Methods supplemental file.

## Supplemental Information

Document S1. Transparent Methods and Figure S1
