# Supplementary material for: Long-Term MALT1 Inhibition in Adult Mice Without Severe Systemic Autoimmunity
Source: iScience. 2020 Sep 12;23(10):101557. doi: 10.1016/j.isci.2020.101557 (PMC7522757; doi:10.1016/j.isci.2020.101557)

iScience, Volume *23*

Supplemental Information

Long-Term MALT1 Inhibition

in Adult Mice Without Severe

Systemic Autoimmunity

Annelies Demeyer, Yasmine Driege, Ioannis Skordos, Julie Coudenys, Kelly Lemeire, Dirk Elewaut, Jens Staal, and Rudi Beyaert

Supplemental Information

Transparent Methods

Mice

Malt1$^{-/-}$, *Malt1*$^{FL/FL}$ and *Malt1*$^{PD/PD}$ C57Bl/6 mice have been described previously (Demeyer et al., 2019). *Malt1*-i-PD mice and control mice were respectively *Malt1*$^{PD/FL}$ and *Mal1t*$^{+/FL}$ C57Bl/6 mice with a tamoxifen-inducible Cre-ERT2 transgene in one *Rosa26* allele (Hameyer et al., 2007), and a LoxP-stop-LoxP (LSL) RFP reporter gene in the other *Rosa26* allele (Luche et al., 2007). All mice were maintained under specific-pathogen free conditions and fed *ad libitum* at the animal house of the VIB/UGent Center for Inflammation Research. Experiments were carried out in accordance with the UGent ethical guidelines and approved by the local ethical committee for mouse experiments of the VIB-Ghent University Faculty of Sciences (approval numbers EC 2015-031, EC2019-081 and EC2019-082). Equal results were obtained with male and female mice unless otherwise specified.

Single cell suspensions and stimulation

Spleens and thymi were mechanically disrupted with the plunger of a syringe and filtered over a 70 µm cell strainer. For the isolation and analysis of DCs, splenocytes were cut in small pieces and incubated at 37°C for 30 minutes in RPMI 1640 containing Liberase TM (Roche, Mannheim, Germany) and DNase I (Roche). All single cell suspensions were subjected to red blood cell lysis with ACK lysis buffer (Lonza).

To assess intracellular cytokine production, spleen single cell suspensions were cultured in complete medium (RPMI 1640 medium supplemented with 10% FCS, sodium pyruvate, L-glutamine, antibiotics and β-mercaptoethanol) and stimulated with PMA (50ng/ml), ionomycin (500ng/ml) and brefeldin A (1µg/ml) for 4 hours at 37°C and 5% CO2.

To assess MALT1 protease activity, spleen single cell suspensions were cultured in complete medium and left unstimulated or stimulated with PMA (200ng/ml) and ionomycin (1µM) for 90 min at 37°C and 5% CO2.

Immunoblot analysis

Splenocytes were lysed in 50 mM Hepes pH 7.6, 250 mM NaCl, 5 mM EDTA, and 0.5% (vol/vol) NP-40, plus phosphatase and protease inhibitors. Lysates were cleared by centrifugation for 15 min at 14,000 rpm and

4°C. Protein concentration was measured by Bradford protein assay (Bio-Rad) and 5x Laemmli buffer (250 mM Tris-HCl pH 8, 10% SDS, 50% glycerol, 0.005% bromophenol blue, 25% β-mercaptoethanol) was added to the lysates. Equal amounts of protein were separated by 8% or 10% SDS–PAGE and analyzed by semi-dry immunoblotting and detection via enhanced chemiluminescence (Perkin-Elmer Life Sciences). The antibodies that were used are anti-BCL10 cleavage-specific (gift from Thijs Baens, Cistim Leuven vzw, Leuven, Belgium), anti-CYLD (sc-74435, Santa Cruz), anti-HOIL1 (sc365523, Santa Cruz), anti-β-actin-HRP (sc-47778, Santa Cruz), HRP-conjugated anti-mouse and anti-rabbit IgG antibody (Thermo Fisher Scientific 31432 and 31464).

Flow cytometry

Splenocytes, thymocytes and blood cells were analyzed with a LSRII or a Fortessa 5 flow cytometer (BD Biosciences) and FlowJo Software (Treestar, Inc, Ashland, Ore) was used for data analysis. Cells were stained for 20 min at 4°C with master mix containing a fixable live/dead dye eFluor 506 or eFluor780, FC block anti-CD16/CD32 and antibodies against surface antigens. Cells were analyzed unfixed or fixed for 30 min at 4°C with the BD Cytofix/cytoperm Kit or the eBioscience Foxp3/Transcription Factor staining buffer set. After fixing, intracellular antigens were stained by adding the corresponding antibodies in the permeabilization solution from the corresponding fixative buffer set for 30 min at 4°C. Antibodies were purchased from BD Biosciences, eBioscience, Thermo Fisher Scientific or Tonbo Bioscience. The following antibodies were used CD3 (145-2C11 or 17A2), CD4 (RM4-5 or GK1.5), CD8 (53-6.7), CD25 (PC61), CD44 (IM7), CD62L (MEL-14), CD11b (M1/70), CD11c (N418), FoxP3 (FJL-16s), TNFR2 (TR75-89), CTLA4 (UC10-4B9), IL-2 (JES6-5H4), IFN-γ (XMG1.2), TNF (MP6-XT22), CD19 (1D3), CD45R/B220 (RA3-6B2), CD21/CD35 (4E3), CD23 (B3B4), Siglec H (440c), Ly6-C (AL-21), NKp46 (29A1.4), Ly6-G (1A8), MHCII (M5/114.15.2), CD172a (P84).

Histology

Stomach, lungs, salivary glands, colon and liver were fixed with 4% paraformaldehyde and imbedded in paraffin. Sections (5 µm) were stained with haematoxylin and eosin. Scoring of immune cell infiltration in lungs and stomach was done blinded. Lung and stomach sections were stained with anti-CD3 (A0452, Dako-Agilent Technologies) or anti-B220 (RA3-6B2, eBioscience) and counterstained with haematoxylin. Images (100x magnification) were acquired with a BX51 discussion microscope (Olympus) using an objective lens with

N.A. 1.0. The 100 µm scale bar was added with Fiji.

Cytokine detection in blood

Blood samples were collected for serum preparation and the levels of IL-5 (171-G5006M), IL-10 (171-G5009M), IL-13 (171-G5012M), IL-17 (171-G5013M), KC (171-G5018M), IFN-γ (171-G5017M) and TNF (171-G5023M) were determined by Bio-Plex (Biorad) according to the manufacturer's conditions.

Statistics

GraphPad Prism 8 software was used for all statistical analysis. Data are represented as means ± SEM. P values were calculated by unpaired Student's t-test, and differences between groups were considered statistically significant with a P value lower than 0.05 or less; $^{*}p < 0.05$, $^{**}p < 0.01$, $^{***}p < 0.001$, and $^{****}p < 0.0001$.

Figure S1. ***Malt1*-i-PD mice have no autoantibodies in their serum after 6 months tamoxifen, Related to Figure 4.** Picture of line immunoassay strips (INNO-LIA ANA Update, Fujirebio) to detect specific antibodies from *Malt1*-i-PD mice. Serum of healthy C57BL/6 mice and serum from a NOD mouse were included as negative and positive controls, respectively. Each strip corresponds to an individual mouse. The assay contains the following recombinant and natural antigens: SmB, SmD, RNP-A, RNP-C, RNP-70k, Ro52/SSA, Ro60/SSA, La/SSB, CenpB, Topo-I/Scl70, Jo-1, ribosomal P, and histones. Nylon strips were incubated with mouse serum at a 1:200 dilution. Following washing, a 1:2,500 dilution of alkaline phosphatase-conjugated anti-mouse IgG was added (Chemicon). After washing, addition of the chromogen 5-bromo-4-chloro-3-indolyl phosphate produced a dark brown color in proportion to the amount of specific autoantibody in the test sample. Sulfuric acid was added to stop the color development.

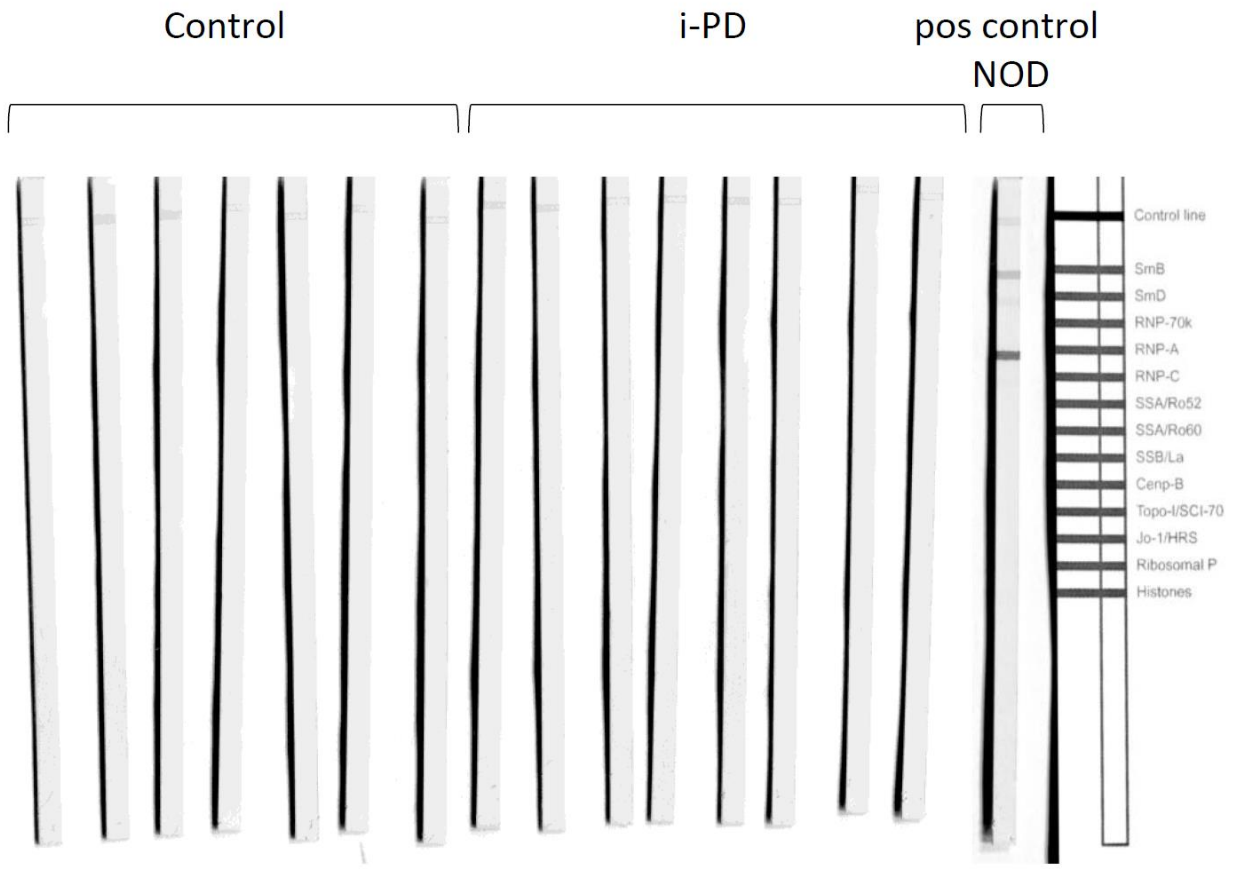

Supplement: Document S1. Transparent Methods and Figure S1 [file mmc1.pdf]
